# Group A streptococci clones associated with invasive infections and pharyngitis in Portugal present differences in *emm* types, superantigen gene content and antimicrobial resistance

**DOI:** 10.1186/1471-2180-12-280

**Published:** 2012-11-27

**Authors:** Ana Friães, Francisco R Pinto, Catarina Silva-Costa, Mario Ramirez, José Melo-Cristino

**Affiliations:** 1 Instituto de Microbiologia, Instituto de Medicina Molecular, Faculdade de Medicina, Universidade de Lisboa, Lisboa, Portugal; 2 Centro de Química e Bioquímica, Faculdade de Ciências, Universidade de Lisboa, Lisboa, Portugal

**Keywords:** Streptococcus pyogenes, Streptococcal M protein, Exotoxins, Pharyngitis, Invasive infection

## Abstract

**Background:**

A few lineages of Group A streptococci (GAS) have been associated with a reemergence of severe invasive streptococcal disease in developed countries. However, the majority of the comparisons between invasive and non-invasive GAS isolates have been performed for collections of reduced genetic diversity or relied on limited typing information to distinguish clones. We characterized by several typing methods and compared a collection of 160 isolates recovered from normally sterile sites with 320 isolates associated with pharyngitis and recovered in the same time period in Portugal.

**Results:**

Although most of the isolates belonged to clones that were equally prevalent in invasive infections and pharyngitis, we identified markers of invasiveness, namely the *emm* types 1 and 64, and the presence of the *speA* and *speJ* genes. In contrast, *emm*4, *emm*75, and the *ssa* and *speL/M* genes were significantly associated with pharyngitis. There was a strong agreement between the *emm* type, the superantigen (SAg) genes and the clusters defined by pulsed-field gel electrophoresis (PFGE) profiling. Therefore, combinations of particular *emm* types and SAg genes frequently co-occurred in the same PFGE cluster, but there was no synergistic or antagonistic interaction between them in determining invasiveness. Only macrolide-susceptible PFGE clones were significantly associated with invasive infections or pharyngitis, while the clones of resistant isolates sharing all other molecular properties analyzed were equally prevalent in the two groups of isolates.

**Conclusions:**

This study confirmed the importance of the widely disseminated *emm*1-T1-ST28 clone in invasive infections but also identified other clones linked to either invasive infections (*emm*64-ST164) or pharyngitis (*emm*4-T4-ST39), which may be more limited in their temporal and geographical spread. Clonal properties like some *emm* types or SAg genes were associated with disease presentation, highlighting the importance of bacterial genetic factors to the outcome of GAS infections, although other, yet unidentified factors may also play an important role.

## Background

*Streptococcus pyogenes* (Lancefield group A *Streptococcus*, GAS) remains one of the most common human pathogens, being responsible for uncomplicated superficial infections of the respiratory tract and skin, such as tonsillo-pharyngitis and impetigo, but also causing severe and rapidly progressing invasive disease such as necrotizing fasciitis, bacteremia, streptococcal toxic shock syndrome (STSS), puerperal sepsis, pneumonia, and meningitis [1]. Although the incidence and severity of GAS infections in industrialized countries decreased for most of the 20^th^ century, a reemerge of GAS invasive disease has been noted since the late 1980s, both in North America and in Europe [2]. The annual incidence of GAS invasive disease has been estimated at 2.45/100 000 for developed countries, with a median case fatality rate of 15% [3].

The increase in the incidence of GAS invasive infections has been frequently associated with specific clones, raising the possibility that the rise of particularly virulent clones was responsible for this reemergence, in particular the M1T1 clone which is dominant among invasive GAS isolates in most developed countries [4,5]. However, a higher representation of a particular clone in invasive infections may be simply due to a high prevalence of that same clone in the general GAS population. To address this question several studies have performed comparisons between the characteristics of the invasive clones and the *S. pyogenes* isolates associated with carriage or uncomplicated infections in the same time period and geographic region. Some of those studies reported a differential distribution of M/*emm* types or of T types between invasive and non-invasive isolates and confirmed an association between serotype T1 and M1/*emm*1 and invasive infection [6-9], but many others found that the major clones responsible for invasive infections had a similar prevalence among non-invasive infections [10-12]. However, most of the studies performing such comparisons were either restricted to small numbers of isolates or were limited in the typing methodologies used, relying essentially on M/*emm* typing.

Serotyping of GAS based on protein M, a major surface virulence factor, has long been used as the gold standard for the epidemiological surveillance of the infections caused by this pathogen. In recent years it has been widely replaced by an equivalent approach based on sequencing the hypervariable region of the *emm* gene encoding the M protein. However, recent studies show that *emm* typing alone is not sufficient to unambiguously identify GAS clones and that it must be complemented with other typing methods such as pulsed-field gel electrophoresis (PFGE) macrorestriction profiling or multilocus sequence typing (MLST) [13]. Streptococcal superantigens (SAgs) secreted by *S. pyogenes* play an important role in the pathogenesis of the infections caused by this species [14]. The profiling of the eleven SAg genes described so far (*speA*, *speC*, *speG, speH*, *speI*, *speJ*, *speK*, *speL*, *speM*, *ssa*, *smeZ*) can be used as a typing methodology [15]. Some studies suggested an association between the presence of certain SAg genes or of certain SAg gene profiles and invasive infections [10,16], although others failed to establish such an association, reporting instead a strong link between the SAg profile and the *emm* type, regardless of the isolation site [12,15].

We have previously characterized a collection of 160 invasive GAS isolates collected throughout Portugal between 2000 and 2005, and found a very high genetic diversity among this collection, but with a dominant clone representing more than 20% of the isolates, which was characterized as *emm*1-T1-ST28 and carried the gene *speA*[17]*.* The aim of the present study was to evaluate if the clone distribution among the invasive GAS isolates in Portugal reflected the clonal structure of the isolates causing pharyngitis, in terms of molecular properties and antimicrobial resistance. In order to do that, 320 non-duplicate isolates collected from pharyngeal exudates associated with tonsillo-pharyngitis in the same time period were studied by *emm* typing, T typing, SAg profiling, PFGE macrorestriction profiling, and selected isolates were also submitted to MLST analysis. All isolates were also tested for their susceptibility to clinically and epidemiologically relevant antimicrobial agents. The great majority of the clones were found with a similar frequency among invasive infections and pharyngitis. Still, some clones were shown to have a higher invasive disease potential and it was also possible to establish significant associations between some *emm* types and SAg genes and disease presentation.

## Results

### Antimicrobial resistance

All isolates were fully susceptible to penicillin, quinupristin/dalfopristin, chloramphenicol, vancomycin, linezolid, and levofloxacin (Table 1). Among the invasive isolates, 19 (12%) were resistant to erythromycin, while the isolates associated with pharyngitis presented significantly higher macrolide resistance – 21% (*P* = 0.016, two-tailed Fisher’s exact test). Among the invasive macrolide-resistant isolates, 10 (53%) presented the M phenotype and were therefore susceptible to clindamycin, whereas the remaining nine (47%) were also constitutively resistant to clindamycin (cMLS_B_ phenotype). The proportion of the two phenotypes was similar among the pharyngitis isolates, with 37 isolates (55%) presenting the M phenotype and 30 (45%) presenting the MLS_B_ phenotype (one with inducible resistance and the others with constitutive resistance to clindamycin). All the isolates presenting the M phenotype of macrolide resistance carried only the *mef*(A) variant of the *mef* determinant. The cMLS_B_ isolates carried only the *erm*(B) gene, except for one pharyngitis isolate which also harbored *mef*(A), and the only iMLS_B_ isolate in the collection that presented the *erm*(A) gene.

**Table 1:** PFGE clusters presenting antimicrobial resistant isolates collected from invasive infections and pharyngitis in Portugal

**PFGE cluster**^***a***^	**Antimicrobial resistance**^***b***^	**No. of resistant isolates**	
		**Invasive**	**Pharyngitis**
C_38_	Tet		1
D_36_	MLS_B_		1
	M		1
G_27_	M	6	19
	M,Tet	1	
H_26_	MLS_B_,Bac	6	17
	Tet		1
I_24_	MLS_B_,Tet		1
J_16_	Tet	12	1
K_14_	M		1
L_13_	MLS_B_,Tet	1	6
	Tet	2	
M_11_	MLS_B_,Tet	1	
N_10_	Tet	1	1
	MLS_B_,Tet		1
O_9_	M	4	5
R_6_	M		3
S_6_	M		1

^*a*^ Clusters are designated by capital letters and a subscript number indicating the number of isolates in each cluster;

^*b*^ The antibiotics tested were penicillin quinupristin/dalfopristin, chloramphenicol, vancomycin, linezolid, levofloxacin, erythromycin, clindamycin, tetracycline, and bacitracin. M, presenting the M phenotype of macrolide resistance; MLS_B_, presenting the MLS_B_ phenotype of macrolide resistance; Tet, non-susceptibility to tetracycline; M,Tet, presenting the M phenotype of macrolide resistance and resistance to tetracycline; MLS_B_,Tet, presenting the MLS_B_ phenotype of macrolide resistance and resistance to tetracycline; MLS_B_,Bac, presenting the MLS_B_ phenotype of macrolide resistance and resistance to bacitracin.

In contrast to erythromycin, tetracycline resistance was much lower among the pharyngitis isolates when compared with the invasive group (6% vs 17%, *P* < 0.001). One invasive isolate presented intermediate resistance to tetracycline (MIC = 3μg/ml). All the resistant strains carried the *tet*(M) gene, except one pharyngitis isolate for which no PCR product was obtained for any of the screened tetracycline-resistance genes. The *tet*(L) gene was detected in only one pharyngitis isolate, which also harbored *tet*(M), while the genes *tet*(K) and *tet*(O) were not amplified in any of the studied isolates. Overall there was a positive association between the genes *tet*(M) and *erm*(B) (*P* < 0.001), but that association was not observed among the subset of invasive isolates, since only three of the 27 *tet*(M)-positive invasive isolates also carried the *erm*(B) gene (*P* = 0.178).

Bacitracin resistance was detected in a total of 23 isolates (5%), with no significant differences among the two types of infection considered. All these isolates expressed the cMLS_B_ phenotype of macrolide resistance and were tetracycline-susceptible.

### Characterization of GAS clones

Globally, among the 480 isolates there were 36 *emm* types, 17 T types, and 49 SAg profiles (the genes included in each SAg profile are presented in Additional file 1). In the subset of 170 isolates (100 from pharyngitis and 70 from invasive infections) selected for MLST analysis, 49 different STs were identified. Nineteen PFGE clusters (groups of > 5 isolates presenting ≥ 80% similarity on the PFGE profile) were obtained including 268 pharyngitis isolates and 143 invasive isolates (86% of all isolates) (Table 2 and Table 3). Except for R_6_, isolates grouped into PFGE clusters presented some variability in their *emm* type, ST, T type, or SAg profile, with most variability found in the later two properties. Still, in most PFGE clusters the majority of the isolates were characterized by a single profile of dominant properties. The *emm* diversity among the PFGE clusters differed significantly (Table 4). Within each PFGE cluster, different *emm* types were associated with distinct SAg profiles (Table 2 and Table 3), although globally the *emm* and PFGE had a similar predictive power over the SAg profile (data not shown).

**Table 2:** Properties of the PFGE clusters with >15 GAS isolates collected from invasive infections and tonsillo-pharyngitis in Portugal

**PFGE cluster**^***a***^	***emm*** **type**	**No. of isolates (% of total)**		**T type**^***b***^**(no. of isolates)**	**SAg genes profile (no. of isolates)**	**ST**^***c***^**(no. of isolates)**
		**Invasive**	**Pharyngitis**			
A_51_	3	15 (9.4)	36 (11.25)	3 (22), NT (14), 3/13 (13), 1 (2)	8 (48), 37 (2), 2 (1)	406 (8), 15 (4), 315 (2)
B_49_	1	28 (17.5)	20 (6.3)	1 (46), NT (2)	10 (47), 3 (1)	28 (10)
	stIL103	1 (0.6)	0	1 (1)	10 (1)	28 (1)
C_38_	89	12 (7.5)	25 (7.8)	B3264 (37)	27 (21), 29 (8), 46 (5), 43 (2), 40 (1)	408 (5), 553 (1), 101 (2)
	75	0	1 (0.3)	25 (1)	42 (1)	150 (1)
D_36_	12	10 (6.3)	25 (7.8)	12 (29), NT (6)	33 (29), 16 (5), 46 (1)	36 (13), 551 (2)
	94	1 (0.6)	0	B3264 (1)	35 (1)	89 (1)
E_30_	6	11 (6.9)	19 (5.9)	6 (27), NT (2), 2(1)	2 (28), 5 (1), 9 (1)	382 (6), 411 (3)
F_29_	4	1 (0.6)	28 (8.8)	4 (29)	23 (27), 22 (2)	39 (5)
G_27_	4	8 (5.0)	19 (5.9)	4 (23), B3264 (2), 2/27/44 (1), 2/4 (1)	23 (23), 30 (2), 40 (1), 41 (1)	39 (8), 561 (1)
H_26_	28	7 (4.4)	17 (5.3)	28 (23), NT (1)	27 (13), 24 (10), 15 (1)	52 (10)
	22	0	1 (0.3)	12 (1)	3 (1)	nd
	75	0	1 (0.3)	NT (1)	7 (1)	481 (1)
I_24_	44/61	6 (3.8)	16 (5.0)	2/27/44 (19), NT (2), 12 (1)	32 (16), 12 (6)	25 (5), 554 (1)
	75	0	1 (0.3)	25 (1)	36 (1)	150 (1)
	89	0	1 (0.3)	5/27/44 (1)	6 (1)	555 (1)
J_16_	64	11 (6.9)	0	3/13 (5), NT (4), 1 (2)	46 (10), 43 (1)	164 (4), 124 (1)
	53	2 (1.3)	0	NT (2)	26 (2)	11 (1)
	74	0	1 (0.3)	B3264 (1)	11 (1)	120 (1)
	87	0	1 (0.3)	28 (1)	38 (1)	62 (1)
	89	0	1 (0.3)	B3264 (1)	43 (1)	568 (1)

^*a*^ Clusters are designated by capital letters and a subscript number indicating the number of isolates in each cluster; ^*b*^ NT, non-typeable; ^*c*^ nd, not determined.

**Table 3:** Properties of the PFGE clusters with <15 GAS isolates collected from invasive infections and tonsillo-pharyngitis in Portugal

**PFGE cluster**^***a***^	***emm*** **type**	**No. of isolates (% of total)**		**T type**^***b***^**(no. of isolates)**	**SAg genes profile (no. of isolates)**	**ST(no. of isolates)**
		**Invasive**	**Pharyngitis**			
K_14_	2	1 (0.6)	13 (4.1)	2 (13), 4 (1)	31 (12), 48 (2)	55 (5)
L_13_	22	1 (0.6)	7 (2.2)	12 (8)	21 (6), 13 (1), 19 (1)	46 (2), 389 (1)
	9	1 (0.6)	1 (0.3)	9 (1), NT (1)	46 (2)	75 (2)
	2	0	1 (0.3)	2 (1)	31 (1)	55 (1)
	74	1 (0.6)	0	9 (1)	5 (1)	120 (1)
	st106M	1 (0.6)	0	4 (1)	49 (1)	53 (1)
M_11_	28	8 (5.0)	3 (0.9)	28 (11)	24 (7), 27 (3), 15 (1)	52 (5)
N_10_	87	2 (1.3)	7 (2.2)	28 (8), 6 (1)	20 (3), 27 (3), 2 (1), 18 (1), 44 (1)	62(2)
	22	0	1 (0.3)	12 (1)	21 (1)	46 (1)
O_9_	1	4 (2.5)	5 (1.6)	1 (8), 13 (1)	10 (9)	28 (4)
P_8_	78	4 (2.5)	4 (1.3)	11 (7), 3/13 (1)	29 (8)	409 (3)
Q_8_	43	4 (2.5)	0	3/13 (2), NT (2)	11 (4)	3 (2)
	58	2 (1.3)	2 (0.6)	NT (4)	17 (3), 14 (1)	410 (3), 176 (1)
R_6_	75	0	6 (1.9)	25 (6)	39 (6)	150 (2)
S_6_	9	1 (0.6)	4 (1.3)	9 (5)	40 (5)	75 (2)
	12	0	1 (0.3)	12 (1)	33 (1)	36 (1)

^*a*^ Clusters are designated by capital letters and a subscript number indicating the number of isolates in each cluster; ^*b*^ NT, non-typeable.

**Table 4:** **Simpson’s index of diversity and 95% Confidence intervals (CI95%) of** ***emm*** **types for each PFGE cluster**

**PFGE cluster**^***a***^	**No.** ***emm*** **types**	**SID [CI**_**95%**_**]**
B_49_	2	0.041 [0–0.118]
C_38_	2	0.053 [0–0.151]
D_36_	2	0.056 [0–0.159]
H_26_	3	0.151 [0–0.336]
I_24_	3	0.163 [0–0.361]
J_16_	5	0.533 [0.255-0.812]
L_13_	5	0.628 [0.353-0.903]
N_10_	2	0.200 [0–0.504]
Q_8_	2	0.571 [0.571-0.571]
S_6_	2	0.333 [0–0.739]

^*a*^ PFGE clusters A_51_, E_30_, F_29_, G_27_, K_14_, M_11_, O_9_, P_8_, and R_6_ include only one *emm* type (SID=0).

Unrelated STs within the same PFGE clusters were associated with isolates of different *emm* types, while isolates of the same *emm* type presented the same ST or single-locus variants (SLVs) (Table 2 and Table 3). The only exceptions were ST39 and ST561 that were both associated with cluster G_27_ and *emm*4, but were double-locus variants (DLVs) of each other. In clone I_24_, four distinct STs were found. While ST25 and ST554 were SLVs and were both associated with *emm*44/61, ST150 belonged to a different clonal complex, but was also associated with a different *emm* type (*emm*75). Finally, ST555 despite being associated with an isolate of a different *emm* type (*emm*89) is a SLV of ST25, which may explain why this isolate was clustered in I_24_ and not in the major PFGE cluster associated with this *emm* type (C_38_).

Isolates expressing the M phenotype of macrolide resistance belonged mostly to clusters G_27_, O_9_, and R_6_, while the majority of MLS_B_ isolates were clustered in H_26_ and L_13_ (Table 1). Isolates belonging to *emm* types 1, 4, and 28 were separated into different PFGE clusters according to macrolide resistance (B_49_ and O_9_ for *emm*1; F_29_ and G_27_ for *emm*4; H_26_ and M_11_ for *emm*28, respectively), while the remaining characteristics (T type, ST, and SAg profile) of each pair of PFGE clusters were the same.

Bacitracin-resistant isolates were all clustered in PFGE H_26_ and were characterized as *emm*28-T28, except for one isolate that was *emm*22-T12. However, this cluster was not restricted to bacitracin-resistant isolates, since it also included three bacitracin susceptible isolates, two of which were also *emm*28-T28, while the other was *emm*75 but T non-typable.

### Surface antigen differences between invasive and pharyngitis isolates

**Figure 1:**
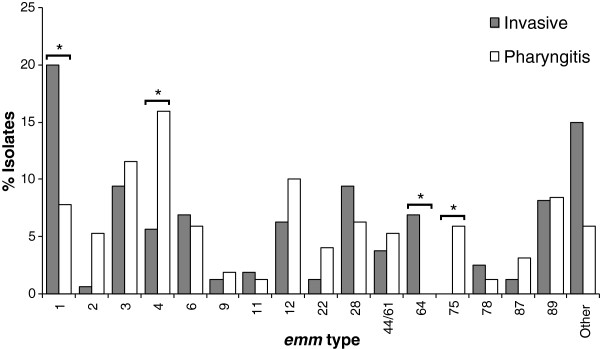
**Distribution of *emm *types among 160 invasive isolates and 320 pharyngitis isolates.** Other includes *emm* types identified in ≤ 5 isolates - *emm*18 (*n*=4), 25 (*n*=1), 29 (*n*=2), 30 (*n*=1), 43 (*n*=4), 48 (*n*=1), 53 (*n*=3), 58 (*n*=5), 74 (*n*=2), 77 (*n*=4), 90 (*n*=1), 94 (*n*=3), 102 (*n*=3), 103 (*n*=1), 113 (*n*=3), 114 (*n*=1), 118 (*n*=1), *st*106M (*n*=1), *st*G1750 (*n*=1), *st*IL103 (*n*=1). The asterisk indicates significant differences (*P*<0.001).

The invasive isolates were significantly less diverse than the pharyngitis isolates by T typing and SAg profiling (Table 5). However, while the *emm* type distribution varied between the invasive and pharyngitis isolates (*P* < 0.001) no differences were noted in the T types. Sixteen *emm* types occurred only in invasive infection or pharyngitis, but in most cases the small number of isolates associated with these *emm* types prevented the differences from reaching statistical significance (Figure 1). In contrast, the overrepresentation of *emm* types 1, 4, 64, and 75 in one of the groups was statistically supported.

**Table 5:** **Simpson’s index of diversity and 95% Confidence intervals (CI**_**95%**_**) of the typing methods used in the analysis of the 160 invasive isolates and 320 pharyngitis isolates**

**Typing method**	**Invasive**		**Pharyngitis**	
	**No. types**	**SID [CI**_**95%**_**]**	**No. types**	**SID [CI**_**95%**_**]**
T typing	13	0.882 [0.859-0.904]	17	0.915 [0.907-0.923]
*emm* typing	30	0.920 [0.900-0.940]	26	0.921 [0.911-0.931]
PFGE profiling	30	0.930 [0.912-0.947]	44	0.947 [0.939-0.954]
SAg profiling	27	0.911 [0.891-0.931]	46	0.941 [0.932-0.951]

### SAg differences between invasive and pharyngitis isolates

A detailed analysis of the SAg profiling results of the isolates is performed elsewhere [18]. Briefly, the chromosomally encoded SAg genes *smeZ* and *speG* were the most frequent among the 480 isolates (*n* = 461 and 417, respectively), followed by *speC* (*n* = 247), *ssa* (*n* = 170), *speJ* (*n* = 157), *speA* (*n* = 154), *speK* (*n* = 118), *speH* (*n* = 82), *speI* (*n* = 73), and *speL* and *speM,* which were always detected together (*n* = 44). The association of individual SAg genes with disease presentation was tested. In the analysis of these results, the SAg genes *speG* and *smeZ* were not considered because they were present in nearly all isolates, and the genes *speL* and *speM* were considered as a single entity, since they always co-occurred. Individually, the genes *speA* and *speJ* were both associated with invasive isolates (*P* < 0.001). As expected, strains carrying both these genes were also associated with invasive infections (*P* < 0.001), but no synergistic effect between the two genes was observed, since the presence of one did not significantly increase the representation of the other among invasive isolates. In contrast, *speC* (*P* = 0.002), *ssa* (*P* < 0.001), and *speL/M* (*P* < 0.001) were individually associated with pharyngitis. The combinations *speC*+*speL/M* and *ssa*+*speL/M* were both associated with pharyngitis (*P* = 0.004 and 0.012, respectively), but there was also no synergistic effect relative to the presence of a single gene. However, the association of *speC* with pharyngitis isolates can be explained by a high frequency of co-occurrence of this gene with *ssa*, since the isolates harboring *speC* without *ssa* were not significantly associated with any of the groups. An interesting situation occurred when analyzing the interaction between *speJ* (associated with invasive infections) and *ssa* (associated with pharyngitis). Among isolates carrying *speJ*, the group that also carried *ssa* was no longer associated with invasive infections, while the association of isolates carrying *ssa* with pharyngitis was not significantly altered by the presence of *speJ*. This argues for a dominant effect of the presence of *ssa* over that of *speJ* in determining the invasive capacity of individual isolates. The association of SAg profiles with disease presentation was also tested. Two SAg profiles presented a significant association with invasive isolates, namely SAg10 (*speA*^+^*speG*^+^*speJ*^+^*smeZ*^+^) and SAg46 (*speG*^+^*smeZ*^+^) (*P* < 0.001). The remaining profiles were not significantly associated with any of the two groups of isolates.

When the same kind of analysis was performed for *emm* types and individual SAg genes, three combinations with statistical significance emerged: the association of isolates presenting *emm*1 and *speA*, and *emm*1 and *speJ* with invasive infections (*P* < 0.001), and the association of isolates carrying *emm*75 and *speL/M* with pharyngitis (*P* = 0.001). In all cases, no synergistic or antagonistic interaction was detected between *emm* type and SAg gene, since the *emm* type did not alter the association of the SAg gene with a particular group of isolates.

### Differences between the PFGE clusters found among invasive infection and pharyngitis

**Figure 2:**
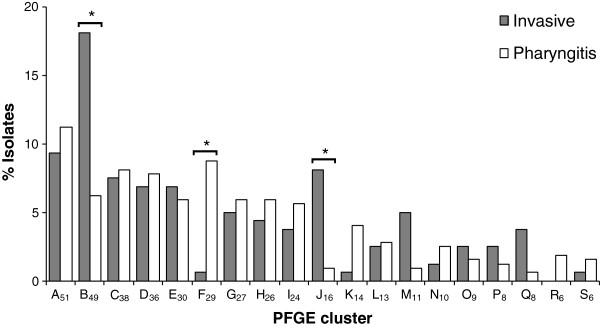
**PFGE clusters found among 160 invasive isolates and 320 pharyngitis isolates.** Approximately 11% of invasive and 16% of non-invasive isolates were included in PFGE clusters of ≤ 5 isolates that are not represented. The asterisk indicates significant differences (*P*<0.001).

The associations described above can be correlated with the PFGE clusters which were also different between the invasive and pharyngitis groups of isolates (*P* < 0.001), in agreement with the differences found in *emm* types (Figure 1 and Figure 2). All the 19 major PFGE clusters occurred in both invasive and pharyngitis isolates, except for R_6_ (*emm*75-T25-ST150-SAg39), which was present only among pharyngeal isolates, but the difference did not reach statistical significance due to the small number of isolates in this cluster. PFGE distinguished several groups of isolates belonging to *emm* types 1 and 4. The difference in the distribution between pharyngitis and invasive infection was not found for all PFGE clusters containing isolates carrying each of these two *emm* types, but only for those including macrolide-susceptible isolates (B_49_ associated with invasive infections and F_29_ associated with pharyngitis, respectively, *P* < 0.001). The *emm*1 and *emm*4 isolates expressing macrolide resistance (M phenotype) were grouped into PFGE clusters O_9_ and G_27_, respectively, which presented a similar prevalence among invasive infections and pharyngitis (Figure 2). PFGE J_16_, which included all *emm*64 isolates, was also associated with invasive infections (P < 0.001). The *emm*75 association with pharyngitis was not translated into an association of a specific PFGE cluster, since the 19 *emm*75 strains were scattered into various PFGE clusters (Table 2 and Table 3).

Not surprisingly, three *emm*-PFGE cluster combinations showed significant associations with infection type: *emm*1-B_49_ and *emm*64-J_16_ were associated with invasive infections, while *emm*4-F_29_ was associated with pharyngitis (*P* < 0.001). It was not possible to detect any synergistic or antagonistic interaction between PFGE and *emm* type in modulating the association of the isolates with either group. The same was true for the statistically significant combinations between PFGE clusters and individual SAg genes, namely the combination of B_49_ with *speA* and with *speJ* (both associated with invasive infections, *P* < 0.001), and the combination of F_29_ with *speC* and with *ssa* (both associated with pharyngitis, *P* < 0.001).

## Discussion

Several studies yielding conflicting results have attempted to compare the clonal composition of GAS populations causing invasive and non-invasive infections in order to identify particularly virulent clones or properties that may be used as epidemiological markers of invasiveness [7,8,11,12,16]. However, many of those studies were limited in the size and diversity of the GAS collections studied or in the typing methodologies used, with most of them relying essentially on *emm* typing, which has been shown to be insufficient for the complete identification of GAS clones [13]. In this work, we used several different typing methods to compare a collection of genetically diverse GAS isolates recovered from normally sterile sites during a period of six years in Portugal [17] with isolates recovered from pharyngeal exudates of patients presenting with tonsillo-pharyngitis, during the same time period and in the same geographical region. The nasopharyngeal mucosa has been suggested to be the main reservoir for GAS isolates associated with invasive infections [19,20].

The differences among the GAS clones associated with invasive infections and pharyngitis were reflected in antimicrobial resistance, with the invasive group of isolates presenting lower macrolide resistance and higher tetracycline resistance, when compared with the pharyngitis group. Among isolates belonging to the same *emm* types, namely *emm*1 and *emm*4, only the macrolide-susceptible clones were associated with either invasive infections or pharyngitis. The macrolide-resistant clones of these *emm* types are reflected in invasive infections according to their prevalence in pharyngitis, suggesting that these are translating more the antibiotic selective pressure than the invasive capacity of the clones. Tetracycline is not currently used in the treatment of GAS infections but resistance to this antimicrobial in *S. pyogenes* is usually acquired by horizontal transfer, since the resistance genes are frequently encoded in mobile genetic elements with a wide host range [21]. These elements often carry macrolide resistance genes as well, and in *S. pyogenes* a significant association between the presence of the genes *erm*(B) and *tet*(M) has been reported and it has been suggested that tetracycline use could contribute to the selection of macrolide-resistant GAS isolates [21,22]. In our study, the association between the presence of the genes *erm*(B) and *tet*(M) was observed globally, but not among the invasive isolates, suggesting that the genetic elements carrying tetracycline resistance conferring genes may be different between the two bacterial populations.

Bacitracin susceptibility is routinely used for the presumptive identification of GAS, although resistant clones have been identified in several countries [23-25]. In our GAS collection, all the bacitracin-resistant isolates (5%), regardless of the type of infection, were clustered in the same PFGE clone (H_26_) and belonged to ST52, although one was *emm*22-T12 while the others were all *emm*28-T28. Isolates with such characteristics had been previously reported in Portugal associated with tonsillo-pharyngitis, skin infections and asymptomatic carriage [26,27], but not with invasive infections. Bacitracin resistance among invasive isolates has been previously reported only among isolates recovered in France and in San Francisco [24,25].

Although 74% of the invasive isolates in our collection belonged to clones which were equally frequent among pharyngitis, suggesting that a significant part of the invasive GAS population mirrors the clonal structure of the circulating GAS isolates, the remaining isolates represented clones that had an enhanced capacity to cause invasive disease. We also found significant associations between individual properties or pairwise combinations of properties and disease presentation. Since in most cases these were also characteristics of the more invasive clones, we cannot exclude that the associations of individual properties or pairwise combinations of properties can reflect, at least partially, the distribution of genetic lineages in the two GAS populations analyzed.

Individually, *emm* types 1 and 64 were associated with invasive infections. Isolates belonging to these *emm* types presented the only two SAg profiles significantly associated with invasive infections. Surprisingly, only one of these SAg profiles includes a phage-encoded SAg gene (*speA*). In agreement with our observation, a previous study found that within the same PFGE-*emm* group, the SAg profiles significantly associated with invasive infections had a smaller number of SAg genes than the dominant profiles in pharyngitis [28]. These results suggest that although some SAg genes may significantly contribute to the virulence of *S. pyogenes*, the rise and success of highly virulent GAS clones may not hinge upon the acquisition of phage-encoded SAg genes. Still, in our study, the SAg genes *speA* and *speJ* were both significantly more prevalent among invasive isolates. This association was not substantially affected by *emm* type, PFGE clone, nor by the presence of other SAg genes, suggesting that *speA* and *speJ* can be regarded by themselves as markers for invasiveness. Although such association has not been previously noted for *speJ*, the *speA* gene has been frequently associated with invasive infections [6,8,16] and the production of SpeA by GAS isolates has been linked to streptococcal toxic shock syndrome [29].

On the other hand, we identified an association of pharyngitis isolates with *emm* types 4 and 75, and with the SAg genes *speC*, *ssa*, and *speL*/*M*. The association of *speC* with non-invasive infections has been previously reported [6,16,30], but in our collection this association could be explained simply by a high frequency of co-occurrence of this gene with *ssa* which was strongly associated with pharyngitis, as was also noted in a recent study [16]. The presence of the genes *speL* and *speM* was not previously associated with non-invasive infections.

Since there is a strong correlation of the SAg profile with *emm* type and of both these properties with PFGE type, some of these individual factors frequently co-occurred in the same clones. Therefore, combinations of these characteristics were also significantly associated with disease presentation. However, we could not detect any synergistic or antagonistic interactions between most of these characteristics, meaning that their co-occurrence in a particular isolate does not make it more invasive than isolates sharing only one of these characteristics.

Two PFGE clusters were significantly more prevalent among isolates associated with invasive disease than among those causing tonsillo-pharyngitis. One of these was a cluster of macrolide-susceptible isolates characterized as *emm*1-T1-ST28 and by the presence of the SAg genes *speA*, *speG*, *speJ*, and *smeZ* (B_49_), which accounted for 18% of the invasive isolates. M1T1 isolates have been frequently associated with severe invasive GAS disease, and the acquisition of prophage-encoded virulence genes, as well as horizontal gene transfer events by homologous recombination were implicated in the increased virulence of these isolates [31,32]. However, many epidemiological studies found a similar prevalence of this clone among invasive and non-invasive isolates [10-12], questioning its enhanced invasive capacity. In contrast to these findings, but similarly to those of others [6-9], we found an association between this clone and invasive GAS disease in Portugal, although it can also frequently cause milder infections such as pharyngitis (it accounted for 6% of the pharyngitis isolates analyzed in this study).

The other cluster significantly associated with invasive infections in Portugal was J_16_, which was dominated by isolates belonging to *emm*64-ST164 and carrying the SAg genes *speG* and *smeZ*. A clone with these characteristics has not been previously associated with invasive disease and *emm*64 has been infrequently reported among invasive GAS isolates [4,33,34]. The higher invasive capacity of this clone cannot be attributed to its SAg repertoire, since these isolates do not harbor any of the SAg genes associated with invasive infection. Other, still unidentified, characteristics may be responsible for the properties of this clone.

In contrast to these PFGE clones, the F_29_ clone of macrolide-susceptible isolates characterized by *emm*4-T4-ST39 and harboring the genes *speC*, *ssa* and *smeZ* was associated with pharyngitis, suggesting that this clone may have a reduced ability to cause invasive disease, in agreement with the negative association between *emm*4 and invasive infection that has been suggested elsewhere [16]. The association of *emm*75 with pharyngitis has not been previously reported and was not translated into particular PFGE clusters due to the high diversity of *emm*75 isolates.

Our data confirms that the widely dispersed M1T1 clone has enhanced invasiveness but we also identified clones with increased or decreased invasive capacity that may have emerged locally and that have a more limited temporal and geographical spread. The *emm* alleles and the SAg genes characteristic of these clones were associated with particular disease presentations. Other individual *emm* alleles and SAg genes were also associated with a higher propensity to cause invasive infections or pharyngitis indicating the importance of these characteristics in determining an isolate’s invasive capacity.

Other factors that were not evaluated in this study may contribute to a different distribution of GAS clones in less severe and more severe infections. These include bacterial factors, such as the occurrence of mutations in transcriptional regulators controlling the expression of virulence factors, which seems to play an important role in the pathogenesis of some GAS isolates [35]. For other clones, the ability to cause invasive infections may be more dependent on exploiting host factors, like the HLA class II haplotype [36], which may vary in frequency in different human populations.

## Conclusions

This study established links between particular genetic lineages and the type of infection, indicating that genetic characteristics of the bacteria play an important role in determining the outcome of their interaction with the human host. The different distribution of clones in the two types of infection supports the relevance of PFGE as a typing methodology for GAS [13]. This was further evidenced by the fact that the macrolide-resistant *emm*1 and *emm*4 PFGE clones were not associated with any particular disease presentation, contrary to the susceptible clones carrying the same *emm* types that were associated with invasive infections and pharyngitis, respectively. Moreover, in contrast to other reports [12,15] we found associations between particular *emm* alleles and SAg genes and disease presentation. In this study, we identified *emm*4, *emm*75, *ssa* and *speL/M* as independent markers for pharyngitis and *emm*1, *emm*64, *speA*, and *speJ* as independent markers for invasiveness. Our data re-enforces the multi-factorial nature of GAS invasive capacity and highlighted lineages and characteristics, in addition to the well known M1T1 lineage, that are associated with particular disease presentations and that may further increase in importance.

## Methods

### Bacterial isolates

The invasive isolates (*n* = 160) were collected from normally sterile sites, and their partial characterization was previously reported [17]. A total of 320 non-duplicate GAS isolates were randomly selected among a collection of 1604 isolates recovered from pharyngeal exudates of patients presenting with tonsillo-pharyngitis in 32 laboratories distributed throughout Portugal, between 2000 and 2005, in the proportion of 1:2 (invasive:pharyngitis) for each studied year. These isolates were recovered from pediatric patients (<18 yrs) and showed a balanced distribution by gender. The subset of macrolide-resistant pharyngeal isolates had been partially characterized [27,37]. Strains were identified by the submitting laboratories and confirmed in our laboratory by colony morphology, β-hemolysis and the presence of the characteristic group antigen (Slidex Strepto A, BioMérieux, Marcy l’Etoile, France).

### Antimicrobial susceptibility testing

Susceptibility tests were performed by disk diffusion on Mueller-Hinton agar supplemented with 5% defibrinated sheep blood, according to the guidelines of the Clinical and Laboratory Standards Institute (CLSI) using the following antibiotic disks (Oxoid, Basingstoke, UK): penicillin, vancomycin, erythromycin, tetracycline, levofloxacin, chloramphenicol, clindamycin, quinupristin/dalfopristin, and linezolid. Whenever isolates with intermediate susceptibility were identified, the results were confirmed by MIC determination using E-test strips (BioMérieux, Marcy l’Etoile, France). The macrolide resistance phenotype was determined as previously described [38]. Susceptibility to bacitracin was determined for all isolates using disks containing 0.05 U of bacitracin (Oxoid, Basingstoke, UK), as described elsewhere [27].

### Detection of antimicrobial resistance genetic determinants

Bacterial DNA was prepared according to the protocols of the Centers for Disease Control and Prevention (CDC) [39]. Determination of the macrolide resistance genotype was performed for strains presenting either the M or the MLS_B_ macrolide resistance phenotype, by a multiplex PCR reaction with primers to detect the *erm*(B), *erm*(A) and *mef* genes, as previously described [40]. Isolates carrying the *mef* gene were subjected to a second PCR reaction in order to discriminate between *mef*(A) and *mef*(E) [37]. Tetracycline resistant isolates were PCR-screened for the presence of the genes *tet*(K), *tet*(L), *tet*(M), and *tet*(O) as previously described [41]. Strains harboring each of the resistance genes were used as positive controls for the PCR reactions.

### T-typing

Strains were cultured in Todd-Hewitt broth (Oxoid, Basingstoke, UK) at 30°C overnight and treated with swine pancreatic extract, using the Auxiliary Reagents for Hemolytic Streptococcus Typing (Denka Seiken, Tokyo, Japan), and following the manufacturer’s instructions. T serotypes were determined by slide agglutination with 5 polyvalent and 19 monovalent sera (Hemolytic Streptococcus Group-A Typing Sera, Denka Seiken).

### *emm*-typing and SAg gene profiling

The *emm*-typing of all isolates was performed according to the protocols and recommendations of the CDC, and the first 240 bases of each sequence were searched against the *emm* CDC database [39]. Identity of ≥ 95% with previously described sequences over the 150 bases considered allowed the assignment of an *emm* type. The presence of the SAg genes *speA*, *speC*, *speG*, *speH*, *speI*, *speJ*, *speK*, *speL*, *speM*, *smeZ*, and *ssa*, and of the chromosomally encoded exotoxin genes *speB* and *speF* (used as positive control fragments) was assessed in all 160 invasive and 320 non-invasive GAS isolates by two multiplex PCR reactions as described elsewhere [18].

### PFGE macrorestriction profiling and MLST

Agarose plugs of bacterial DNA were prepared as previously described [27]. After digestion with SmaI or Cfr9I (Fermentas, Vilnius, Lithuania), the fragments were resolved by PFGE [27]. The isoschizomer Cfr9I was used only for the isolates with the M phenotype, which were not digested by SmaI [13,27]. The macrorestriction patterns generated were compared using the Bionumerics software (Applied Maths, Sint-Martens-Latem, Belgium) to create UPGMA (unweighted pair group method with arithmetic mean) dendrograms. The Dice similarity coefficient was used, with optimization and position tolerance settings of 1.0 and 1.5, respectively. PFGE clones were defined as groups of >5 isolates presenting profiles with ≥ 80% relatedness on the dendrogram [13]. MLST analysis was performed as described elsewhere [42] for representatives of each PFGE cluster (a total of 100 non-invasive and 70 invasive isolates). When more than one *emm* or T-type was present in the same PFGE cluster, isolates expressing different surface antigens were selected. Allele and sequence type (ST) identification was performed using the *S. pyogenes* MLST database [43]. Whenever new alleles were identified, chromatograms of both strands were submitted to the database curator for approval and an allele number was assigned. Clonal complexes were determined using the goeBURST algorithm implemented in PHYLOViZ [44].

### Statistical analysis

The diversities of the different PFGE clusters were compared using the Simpson’s index of diversity (SID) with corresponding 95% confidence intervals (CI_95%_) [13]. Differences in antibiotic resistance between the invasive and non-invasive groups of isolates were evaluated using Fisher’s exact test. *P* values < 0.05 were considered to indicate statistical significance. SAg genes, *emm* types and PFGE types were screened for associations with the invasive group by computing an odds-ratio and an associated Fisher’s exact test. Additionally, pairs of individual SAg genes with each other or with *emm* types or PFGE types were similarly tested for the association of each pairs’ co-occurrence with the invasive group of isolates. For the pairs where at least one of the types individually or their co-occurrence were associated (either positively or negatively) with the invasive group, two more tests were done, to investigate if the association of one of the types individually was modified by the co-occurrence of the other type in the pair (synergism or antagonism). Considering a pair of types A and B, this test compares the proportion of invasive isolates among the ones that have A type but not B with the same proportion among isolates that have both A and B types. If the proportions are statistically different, according to a Fisher’s exact test, we can conclude that type B modifies the association of type A with the invasive group of isolates. Conversely, if the proportion of invasive isolates among the ones that have the B type but not A differs from the same proportion among isolates that have both A and B types, type A modifies the association of type B with the invasive group. If the isolates that are simultaneously of the A and B type show a significantly stronger association with invasive infection than the one observed for isolates having either the A or B type, the types are said to be synergistic. If, on the other hand, isolates that are simultaneously of the A and B type show a significantly weaker association with invasive infection than the one observed for isolates having either the A or B type, the types are said to be antagonistic. All the p-values obtained in each step of the screening procedure were corrected for multiple testing through the False Discovery Rate (FDR) linear procedure [45].

## Competing interests

Dr José Melo-Cristino has received research grants administered through his university and received honoraria for consulting and serving on the speakers bureaus of Pfizer, Bial, GlaxoSmithKline and Novartis. Dr Mário Ramirez has received honoraria for consulting and serving on speakers bureau of Pfizer. The other authors declare no conflict of interest. The funders had no role in study design, data collection and analysis, decision to publish, or preparation of the manuscript. This work was partially supported by Fundação para a Ciência e Tecnologia, Portugal (PTDC/SAU-ESA/72321/2006), Fundação Calouste Gulbenkian and unrestricted research grant from Glaxo SmithKline.

## Authors’ contributions

AF, CSC performed the majority of the experiments. AF, MR and JMC have made substantial contributions to conception and design. AF, FRP and MR analysed and interpreted the data. All authors have been involved in drafting the manuscript and revising it critically for important intellectual content. All authors read and approved the final manuscript.

## Supplementary Material

Additional file 1 SAg genes profiles identified in GAS isolates in Portugal. Click here for file
