# Supplementary material for: Group A streptococci clones associated with invasive infections and pharyngitis in Portugal present differences in emm types, superantigen gene content and antimicrobial resistance
Source: BMC Microbiol. 2012 Nov 27;12:280. doi: 10.1186/1471-2180-12-280 (PMC3543706; doi:10.1186/1471-2180-12-280)
Supplement: Additional file 1 — SAg genes profiles identified in GAS isolates in Portugal. [file 1471-2180-12-280-S1.pdf]

Additional file 1 – SAg gene profiles identified in GAS isolates in Portugal.

SAg profile	No. of isolates	*speA*	*speC*	*speG*	*speH*	*speI*	*speJ*	*speK*	*speL*	*speM*	*ssa*	*smeZ*
1	1	+	+	-	-	-	-	-	-	-	+	+
2	31	+	+	+	-	-	-	+	-	-	-	+
3	2	+	+	+	-	-	+	-	-	-	-	+
4	2	+	+	+	-	-	-	-	+	+	-	+
5	2	+	+	+	-	-	-	-	-	-	-	+
6	1	+	-	+	+	+	+	-	-	-	+	+
7	1	+	-	+	+	-	+	-	-	-	-	+
8	49	+	-	+	-	-	-	+	-	-	+	+
9	1	+	-	+	-	-	-	+	-	-	-	+
10	57	+	-	+	-	-	+	-	-	-	-	+
11	7	+	-	+	-	-	-	-	-	-	-	+
12	6	-	+	+	+	+	+	-	-	-	+	+
13	3	-	+	+	+	+	-	-	-	-	+	+
14	1	-	+	+	+	-	-	-	-	-	+	+
15	2	-	+	+	+	+	+	+	-	-	-	+
16	15	-	+	+	+	+	-	-	-	-	-	+
17	3	-	+	+	+	-	-	-	-	-	-	+
18	1	-	+	+	-	-	+	+	-	-	+	+
19	3	-	+	+	-	-	-	+	-	-	+	+
20	5	-	+	+	-	-	+	-	-	-	+	+
21	7	-	+	+	-	-	-	-	-	-	+	+
22	3	-	+	-	-	-	-	-	+	+	+	+
23	52	-	+	-	-	-	-	-	-	-	+	+
24	17	-	+	+	-	-	+	+	-	-	-	+
25	2	-	+	+	-	-	-	+	+	+	-	+
26	2	-	+	+	-	-	-	+	-	-	-	+
27	44	-	+	+	-	-	+	-	-	-	-	+
28	3	-	+	+	-	-	-	-	+	+	-	+
29	21	-	+	+	-	-	-	-	-	-	-	+
30	3	-	+	-	-	-	-	-	-	-	-	+
31	16	-	+	+	-	-	-	-	+	+	-	-
32	16	-	-	+	+	+	+	-	-	-	+	+
33	30	-	-	+	+	+	-	-	-	-	-	+
34	1	-	-	+	+	-	+	-	-	-	-	+
35	3	-	-	+	+	-	-	-	-	-	-	+

SAg profile	No. of isolates	*speA*	*speC*	*speG*	*speH*	*speI*	*speJ*	*speK*	*speL*	*speM*	*ssa*	*smeZ*
36	1	-	-	+	-	-	-	+	+	+	+	+
37	2	-	-	+	-	-	-	+	-	-	+	+
38	2	-	-	+	-	-	+	-	-	-	+	+
39	8	-	-	+	-	-	-	-	+	+	+	+
40	8	-	-	+	-	-	-	-	-	-	+	+
41	1	-	-	-	-	-	-	-	-	-	+	+
42	2	-	-	+	-	-	-	+	+	+	-	+
43	5	-	-	+	-	-	-	+	-	-	-	+
44	2	-	-	+	-	-	+	-	-	-	-	+
45	5	-	-	+	-	-	-	-	+	+	-	+
46	26	-	-	+	-	-	-	-	-	-	-	+
47	2	-	-	-	-	-	-	-	-	-	-	+
48	2	-	-	+	-	-	-	-	+	+	-	-
49	1	-	-	-	-	-	-	-	-	-	-	-
